# Belief or disbelief in feedback influences the detection efficiency of the feedback concealed information test

**DOI:** 10.3389/fpsyg.2022.983721

**Published:** 2022-08-25

**Authors:** Jiayu Cheng, Yanyan Sai, Jinbin Zheng, Joseph M. Olson, Liyang Sai

**Affiliations:** ^1^Department of Psychology, College of Education, Hangzhou Normal University, Hangzhou, Zhejiang, China; ^2^Mental Health Counseling Center, Zhejiang University of Science and Technology, Jinhua, China; ^3^Department of Psychology, Northwestern University, Evanston, IL, United States

**Keywords:** belief, concealed information, deception, feedback, FRN, P300

## Abstract

The feedback concealed information test (fCIT) is a new variant of the CIT that added feedback about participants’ concealing performances in the classical CIT. The advantage of the fCIT is that the resulting feedback related event-related potentials (ERPs) can be used to detect concealed information. However, the detection efficiency of feedback-based ERPs varies across studies. The present experiment examined whether the extent participants believed the feedback influenced their detection efficiency. Specifically, participants did a mock crime and were then tested in a fCIT. Following the fCIT, participants were asked to report how much they believed the feedback was accurate. Results showed that there were no significant correlations between the amplitude of the feedback related negativity (FRN), feedback P300, and participants’ self-report at the group level. However, individual analyses showed that the detection efficiency of both the FRN and feedback P300 were influenced by participants’ belief about the presented feedback. The detection efficiency of the FRN and the feedback P300 was higher among participants who believed the feedback. These findings suggest that the fCIT is dependent to some extent on the participants’ level of belief in the feedback.

## Introduction

Terrorism has become a quite serious problem in modern times. For example, the Boston Marathon bombing on April 15, 2013, caused three deaths as well as 264 injuries. If law enforcement could screen out suspects during the security check process and use lie detection technologies to catch terrorists, the occurrence of such incidents would sharply decline. Therefore, it is important to develop reliable tools to detect deception or concealed information.

The concealed information test (CIT) was proposed by Lykken (1959) to use physiological responses to detect crime-related information in a suspect’s memory (see Ben-Shakhar, 1977; Meijer et al., 2014). In a typical CIT test, participants are presented with a series of items, one of which is the crime-related information (also called probe, e.g., knife), and the other are crime-unrelated items (also called irrelevants, e.g., gun, brick, ice pick, and rope). Guilty participants recognize the probe uniquely among the various irrelevant items and consequently exhibit different physiological responses to it, while innocent participants cannot distinguish between probes and irrelevants, and therefore show similar physiological responses to all items (Klein Selle et al., 2016, 2017, 2019).

With the development of neuroscience technologies such as event-related potential (ERP) and functional magnetic resonance imaging (fMRI), researcher have begun to combine the CIT with brain activity to detect concealed information. Among the ERP-based CIT literature, the P300 is intensively studied (for review, see Rosenfeld, 2019). P300 is a positive wave that typically appears 300–800 ms after stimulus presentation and is often evoked by rare, familiar, and meaningful stimuli (Johnson, 1986; Polich, 2007). In a P300-based CIT, researchers compare the P300 amplitude evoked by the probe and irrelevants. Because the probe is of great significance to guilty participants (Rosenfeld et al., 2012), they produce a larger P300 wave in response to the probe compared to irrelevants. It is on the basis of the probe-irrelevant difference that “guilty” or “innocent” classifications can be made for a given subject in the P300-based CIT.

To further improve detection efficiency, researchers have begun to explore additional ERP components that detect concealed information independently of the P300. For example, Gamer and Berti (2010) found that N200, which is thought to be associated with cognitive control and conflict monitoring (Kopp et al., 1996; Hu et al., 2011), can be used to detect concealed information, because the act of concealing requires participants to monitor conflict with the truthful state (also see Hu et al., 2011; Rosenfeld et al., 2019). Sai et al. (2016) introduced a new variant of the CIT that provides participants with feedback on their concealment performance for each trial (also known as the fCIT). By this design, they not only can use the so-called recognition-related P300 (evoked by probes and irrelevants) but can also use feedback-related ERP potentials (evoked by feedback stimuli) to detect concealed information. In their studies, they focused on two feedback-related potentials: feedback-related negativity (FRN) and feedback-P300. FRN is a negativity that typically appears 250–350 ms following the presentation of feedback stimuli, and previous studies have shown that negative feedback such as monetary loss (e.g., failure) evoked a more negative FRN than positive feedback, such as monetary gain (e.g., Gehring and Willoughby, 2002; Yeung and Sanfey, 2004; Di Gregorio et al., 2019). However, recent studies that used temporal principal component analysis (PCA) to extract FRN found that the FRN reflects a reward-related positivity that is either absent or suppressed following monetary loss (Foti et al., 2011). The feedback-P300 is a positive waveform that appears around 300–500 ms after the feedback stimulus. Despite some previous studies suggesting that there is separation in the functionality of feedback processing (Yeung and Sanfey, 2004), other studies revealed that both FRN and feedback-P300 are sensitive to motivation (Sato et al., 2005; Yeung et al., 2005; Peterburs et al., 2013). Since guilty participants are typically motivated to conceal their knowledge of the probe (e.g., to avoid getting “caught”), feedback following probe would evoke larger FRN and feedback-P300 amplitudes compared to irrelevants among guilty participants. In contrast, feedback following probe would evoke the same FRN and feedback P300 among innocent participants because they are not aware of the probe and therefore have no motivation to conceal it.

Consistent with this hypothesis, researchers have consistently found that feedback-related ERPs can distinguish guilty from innocent participants (Sai et al., 2014, 2016). However, despite this promising initial findings, detection efficiency varies across studies. For example, Sai et al. (2016) found that the FRN can effectively distinguish guilty participants from innocent participants with a high detection efficiency (AUC = 0.95). However, Zheng et al. (2022) reported lower detection efficiency using FRN (AUC = 0.70). Furthermore, Sai et al. (2020) later found that the FRN could not distinguish between guilty and innocent significantly above chance (AUC = 0.53). Moreover, for the feedback P300, Sai et al. (2016) found that the feedback P300 could significantly distinguish between guilty and innocent with high efficiency (AUC = 0.97), In the following two studies, the reported discrimination efficiency of the feedback P300 was also lower (AUC = 0.91, Sai et al., 2020; AUC = 0.80, Zheng et al., 2022). These divergent findings raise an important question about which factors influence the detection efficiency of feedback-related ERPs. One possible factor that could explain these divergences is how much participants believe that the feedback is based on their actual performance. Importantly, although participants are told that the provided feedback is based on their performance during the CIT, the feedback is in fact presented randomly. Thus, it is possible that some participants may disbelieve the feedback, thus decreasing their motivation to conceal information and consequently resulting in decreased detection efficiency when using feedback-related ERPs (Yeung et al., 2005).

Taken together, the current study has two aims. The first aim is to replicate the findings about detection efficiency when using feedback-related potentials to detect concealed information. The second aim is to test whether the detection efficiency of feedback related ERPs is influenced by how much participants believed the feedback we provided. Specifically, participants were asked to do a mock crime and then instructed to conceal crime-relevant information during the fCIT. After the fCIT, participants were then asked to report how much they thought the feedback provided was based on their actual performance. Based on the previous findings, we predict that feedback following the probe will induce greater FRN and fP300 than irrelevants among the guilty, but there will be no difference among the innocent. Given that feedback-related ERPs are sensitive to motivation (Yeung et al., 2005), and disbelief in feedback may hurt participants’ motivation, we expected that detection efficiency of feedback-related ERPs would be better among participants who believed the feedback compared to participants who did not believe the feedback.

## Materials and methods

### Participants

We recruited 60 participants from Hangzhou Normal University. Six participants were excluded from further analyses due to a high number of artifacts in their EEG data, and another two participants were also excluded because they did not fill out the questionnaire. The final sample size was 52 participants (13 male, Mean age = 21.46 years, *SD* = 1.82 years), 24 in the guilty group, and 28 in the innocent group. All participants had normal or corrected vision and were right-handed. At the end of the experiment, each participant got a payment of 60 RMB (equivalent to approximately 9.18 USD). The study was approved by the ethics committee of Hangzhou Normal University.

### Procedures

#### Mock crime phase

After coming into the lab, participants signed the informed consent, and then they were randomly assigned to the guilty group or the innocent group. Participants assigned to the guilty group were told, “Now you are assigned to the guilty group. There’s a valuable item (*ring*) in this room. It’s in an envelope. You need to steal it.” Participants assigned to the innocent group were only asked to visit the room without committing any mock crime. In addition, we put a *wallet* on the table in the room so that all participants could see it. And we used the *wallet* as the target in the subsequent feedback concealed information test. To ensure that each participant saw the *wallet*, after the participants left the room, the experimenter asked participants whether they saw a *wallet* on the table and what color it was. If the participant reported that he did not see the wallet, the participants would return to the room to be sure that they saw the *wallet*.

#### Feedback-CIT phase

After the mock crime task, the participants were taken to the EEG laboratory. During the fCIT, each of the six stimuli was presented to the participants one by one. If participants recognized the item, they were instructed to press the “F” key. If they did not recognize the item, they were instructed to press the “J” key. We balanced the keys between the participants. Since all participants were exposed to the target (*wallet*), all participants were asked to press the “F” key, indicating “Yes, I know this item.” For the four unrelated items (*watch, necklace, bracelet,* and *earrings*) none of the participants were knowledgeable of them, so they were instructed to press the “J” key, which means “No, I do not know this item.” In addition, we further instructed the guilty to conceal their knowledge of the *Ring* (probe) and asked them to press the “J” key.

Feedback was presented following the participant’s response to each stimulus. Participants were told that the feedback indicated the outcome of the brainwave test. There were two kinds of feedback: “+ 4” means “you were telling the truth,” and “– 2” means “you were telling a lie.” In truth, only the feedback following the target was based on the participants’ actual response, while the feedback following the probe and irrelevants were presented randomly (but the participants did not know this). The purpose of choosing “+ 4″ and “– 2” as feedback was to make the subjective value of gain and loss equal (Tversky and Kahneman, 1992; Bress and Hajcak, 2013).

**Figure 1:**
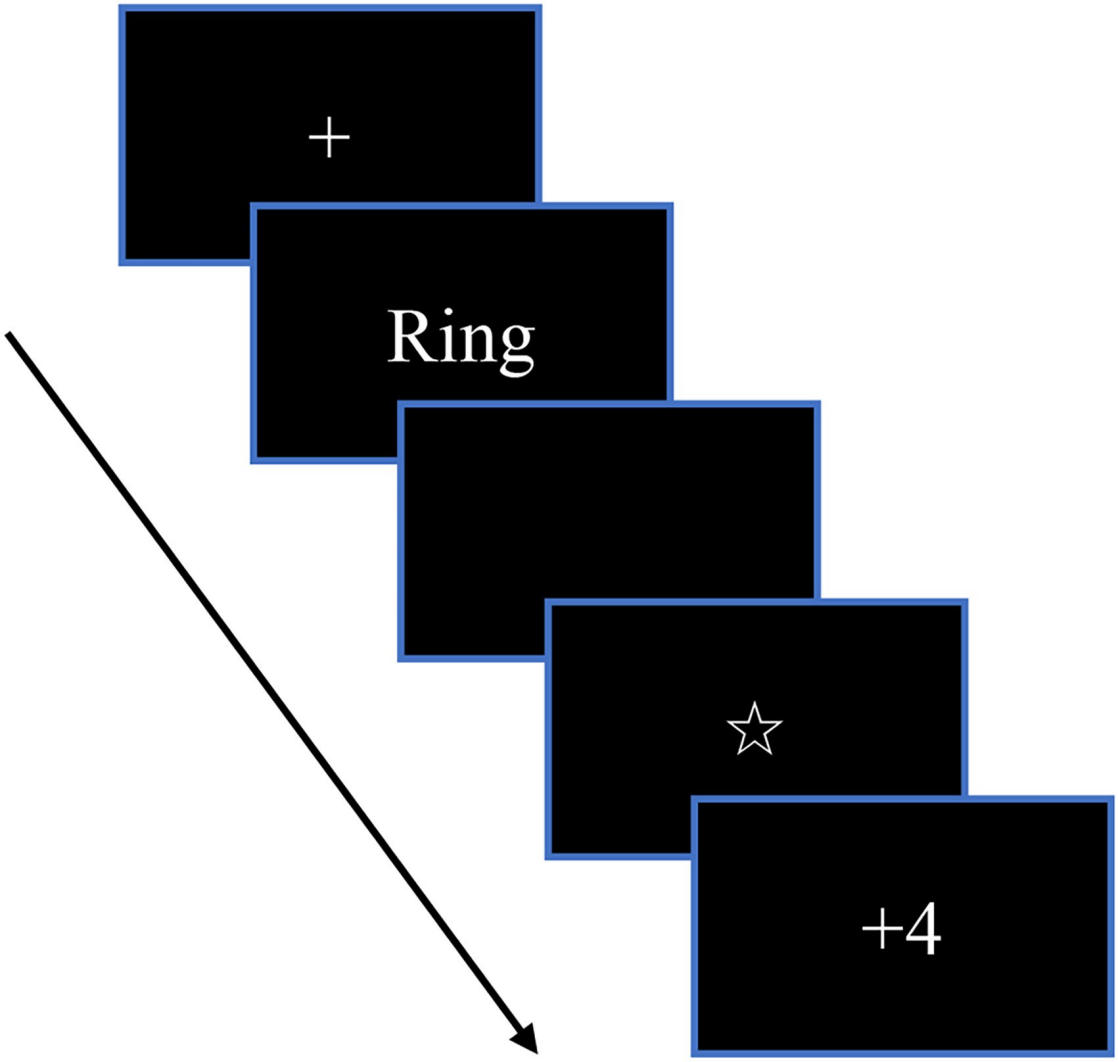
Task structure, Feedback Concealed Information Test.

All the participants sat about 1 m away from the computer. Each stimulus was presented in white font on a black background. Each trial started with a fixed point of 500 ± 100 ms long and then randomly presented one of six stimuli (*wallet, ring, bracelet, earring, necklace*, *and watch*) in the middle of the screen. Each stimulus was presented for 300 ms, and then there was a black screen of 1,000 ms. Participants were instructed to respond as soon as possible when they saw the stimuli (See Figure 1). After participants made responses, a pentagram (“☆”) appeared on the screen for 2,500 ms, which indicated that the lie detector was analyzing the participants’ brainwaves. Then feedback “+ 4” or “– 2” was shown on the screen for 1,000 ms. Each stimulus repeated 60 times, and thus there were 360 trials in total. Participants could have a rest every 40 trials, and the whole task lasted about 40 min.

After the main experiment, participants were asked to report how much they thought the “–2” and “+4” feedback given by the lie detector was accurate on a five-point scale (not accurate at all) to 5 (extremely accurate).

### EEG acquisition

According to the international 10–20 system, EEG was recorded by the actiCAP system (Brain Products, Germany) with 32 Ag/AgCl electrodes and monitored by the BrainVision recorder software. On-line recordings were referenced to right mastoids. Electrode impedance was kept below 10 kΩ, the sampling rate was set to 1,000 Hz.

For off-line analyses, EEG data were processed with BrainVision Analyzer software 2.1 (Brain Products, Germany). Firstly, the raw data were processed by the Ocular Correction ICA method to remove eye movements, then the artifacts were removed by Raw Inspection. And continuous EEGs in recognition stage were filtered with a 6 Hz low-pass filter and a 0.16 Hz high-pass filter (Rosenfeld et al., 2013; Rosenfeld, 2019). Then the continuous EEG records were further segmented into epochs of 1,500 ms duration, included a 200 ms pre-stimuli baseline and 1,300 ms time window after the stimulus presentation. Epochs were baseline-corrected and trials containing signals exceeding ±100 μV were defined as artifact trials and thus excluded from further analyses. Then, the data were averaged across condition, and ERPs in response to irrelevant stimuli were averaged across all four irrelevant items (Also see, Winograd and Rosenfeld, 2014; Olson et al., 2019).

We used the “peak-to-peak” (p–p) method to analyze the recognition-P300. The p–p method searched for the maximally positive mean 100 ms segment from 300 to 800 ms and used the midpoint as P300 latency. It then searched for the maximally negative mean 100 ms segment following recognition-P300 latency and up to 1,300 ms. The “peak-to-peak” value is the amplitude difference between the mean maximally positive segment and the mean maximally negative segment (also see, Olson et al., 2018).

For the feedback stage, we focused on FRN and the feedback P300. Since FRN and the feedback P300 are greatly overlapped, a temporal PCA was conducted in order to score them using the ERP PCA toolkit, version 2.86 (Dien, 2010). Before conducting the PCA, continuous EEG was filtered using a 30 Hz low-pass and 0.1 Hz high-pass. These filter settings were chosen because previous studies have shown that the FRN is composed of beta and theta, whose frequency range is from 6 to 30 Hz, and the feedback P300 is composed of delta, whose frequency range is from 0 to 6 Hz (Li et al., 2016). Then the continuous EEG was further segmented into epochs of 1,200 ms duration, which contains a 200 ms baseline before the feedback stimulus presentation (200 ms) and 1,000 ms time window after the stimulus presentation. Trials with signals exceeding ±100 μV were defined as artifact trials and were excluded from averaging. After baseline correction, the total average processing was performed. The number of valid trials included in each condition is shown in Table 1.

**Table 1:** The number of averaged trials (SE) in each condition.

	Guilty	Innocent
Probe	51.50 (1.27)	50.57 (1.26)
Irrelevants	207.71 (4.82)	200.50 (4.30)
Probe-success	25.54 (0.69)	25.00 (0.77)
Probe-failure	25.87 (0.60)	25.21 (0.70)
Irrelevants-success	104.33 (2.35)	100.75 (2.05)
Irrelevants-failure	102.96 (2.60)	99.54 (2.04)

The PCA was conducted using combined guilty and innocent group data. The Temporal PCA performed the promax rotation (Dien et al., 2007), using 1,200 time points of the average ERP of each subject as variables, participants, and conditions as observation values. According to the scree plots, a total of 13 factors can be extracted, of which seven factors account for more than 1% of the total variance of the data, which meets the standard. Given the fact that the FRN always occurs between 250 and 350 ms, and the feedback-P300 always occurs between 400 and 600 ms, we chose the positive component that reached the peak at 238 ms as FRN, and the positive component that occurred at 416 ms as feedback-P300. Table 2 lists the PCA factors selected for statistical analysis.

**Table 2:** PCA factors selected for statistical analysis.

Corresponding ERP component	Temporal factors	Temporal loading peaks (ms)	Variance explained (%)
FRN	TF03	230 ms	4.04%
Feedback-P300	TF01	390 ms	12.84%

### Statistical analysis

All analyses were performed with SPSS 22.0. In the ANOVA, when the assumption of sphericity was violated, the Greenhouse-Geiser correction was applied. Fisher’s least significant difference was used to calculate *post hoc* comparisons. The effect size of significant effects was reported as partial eta squares and Cohen’s *d*.

In addition to the classical statistical inference, we use Jeffreys-Zellner-Siow (JZS) Bayes factors (BFs; scale *R* = 0.707; see Rouder et al., 2009) as an alternative and/or supplementary statistical method. BFs are an important method for model comparison and hypothesis testing in Bayesian statistics. It can be interpreted as the degree of support for null hypothesis or alternative hypothesis (Kass and Raftery, 1995; Jeffreys, 1998). In this study, we reported BF_10_ (favoring the alternative hypothesis) or BF_01_ (favoring the null hypothesis) for *t*-tests, and BF_Inclusion_ (favoring the model containing the effect of interest) or BF_Exclusion_ (favoring the model absent the effect of interest) for ANOVAs. The BF value of ≥3 was regarded as moderate evidence for the respective hypothesis (Kass and Raftery, 1995). The BFs in this study were calculated by the open software JASP (Version 0.14.1, https://jasp-stats.org/, JASP Team, 2020).

Lastly, receiver operating characteristic (ROC) analyses were conducted to examine the detection efficiency of each ERP component. The ROC curve is a comprehensive evaluation method used to describe and compare diagnostic tests (McClish, 1989). The composition of the ROC curve can reflect the relationship between sensitivity and specificity at different cutpoints, and the efficiency of the diagnostic test can be judged by calculating the area under ROC curve (AUC). The ROC analyses in the present study were conducted based on the probe minus irrelevants P300, FRN, and feedback-P300 in each group. The ROC analysis in this study takes the amplitude difference of each ERP component (P300, FRN, and feedback P300) in the probe minus irrelevants as the test variable, the guilty group as the state variable of the analysis. The joint indicators were tested by converting each index into a *Z* score and adding the data as test variables.

## Results

### Recognition-P300

**Figure 2:**
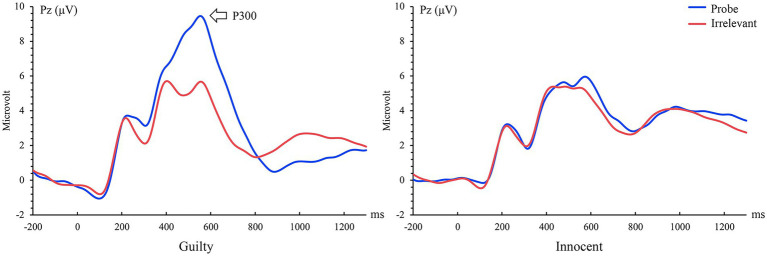
Grand-average probe/irrelevant evoked event-related potential (ERP) waveforms from Pz.

A two-way 2 stimulus type (probe vs. irrelevant) by 2 group (guilty vs. innocent) mixed ANOVA was conducted with P300 amplitude as the dependent variable. Results showed a significant main effect of stimulus type, *F* (1, 50) = 19.65, *p* < 0.001, *η_p_*^2^ = 0.28, BF_Inclusion_ = 1.70 × 10^4^, the probe elicited larger P300 amplitude than the irrelevants (7.50 ± 0.73 vs. 4.28 ± 0.40 μV); The main effect of group was also significant, *F* (1, 50) = 12.19, *p* = 0.001, *η_p_*^2^ = 0.20, BF_Inclusion_ = 3.79 × 10^3^, the P300 in the guilty group was significantly larger than that in the innocent group (7.52 ± 0.69 vs. 4.26 ± 0.63 μV); The interaction between group and stimulus type was also significant, *F* (1, 50) = 17.27, *p* < 0.001, *η_p_*^2^ = 0.26, BF_Inclusion_ = 1.12 × 10^3^. *Post hoc* test showed that among guilty participants, probe elicited significantly larger P300 than irrelevants (10.63 ± 1.07 vs. 4.40 ± 0.59 μV, *t*(23) = 5.85, *p* < 0.001, Cohen’s *d* = 1.14, BF_10_ = 4.75 × 10^3^); while there was no significant difference between the probe and the irrelevants in the innocent group (4.36 ± 0.99 vs. 4.16 ± 0.55 μV, *t*(27) = 0.20, *p* > 0.05, Cohen’s *d* = 0.07, BF_01_ = 4.74). Brainwave forms of guilty and innocent group in the recognition stage are shown in Figure 2.

### FRN at Fz (230 ms)

**Figure 3:**
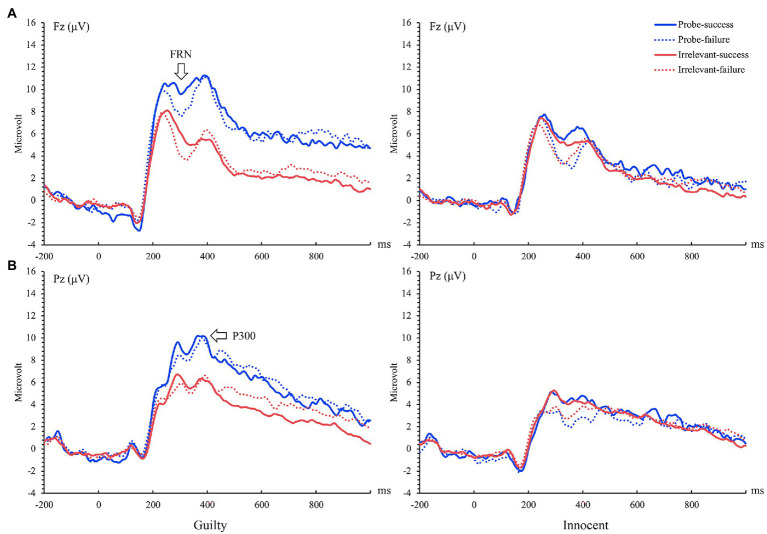
Grand-average ERP waveforms before principal component analysis (PCA) transformation of feedback related negativity (FRN) **(A)** and feedback-P300 **(B)** induced by guilty and innocent in the feedback stage.

**Figure 4:**
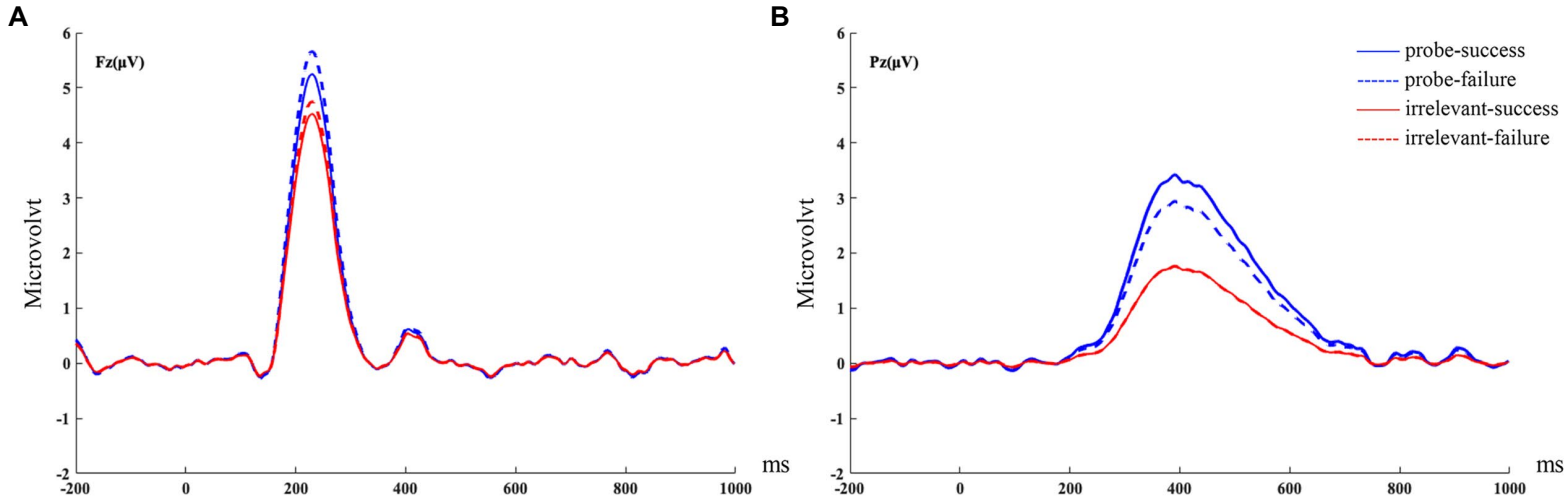
PCA-extracted ERP waveforms of FRN **(A)** and feedback-P300 **(B)** during feedback stage combining guilty and innocent group.

**Figure 5:**
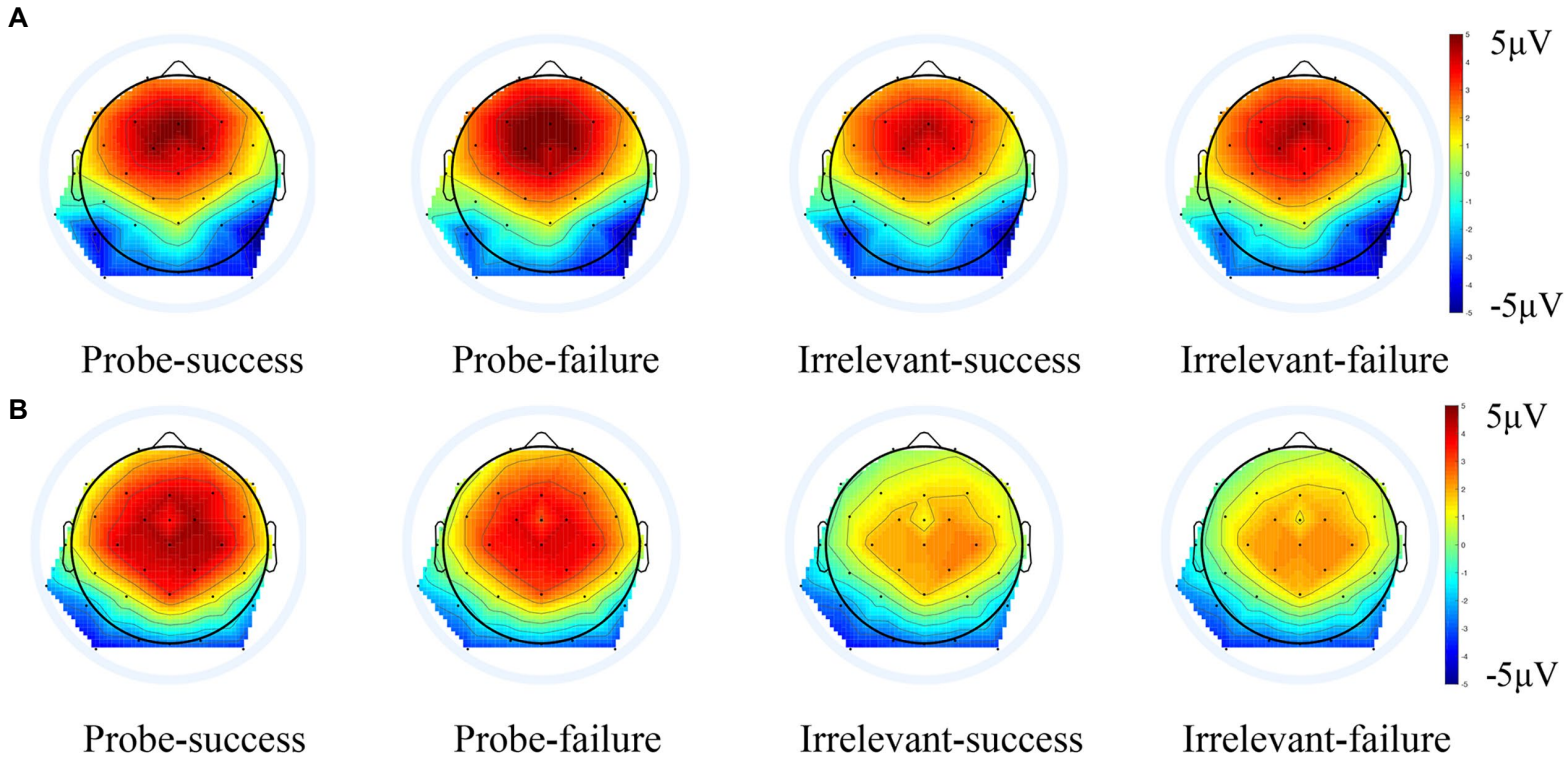
PCA-based Scalp distribution of the FRN **(A)** and feedback-P300 **(B)** during feedback stage combining guilty and innocent group.

We analyzed the amplitude of FRN by using a mixed ANOVA of 2 groups (guilty vs. innocent) × 2 stimulus type (probe vs. irrelevants) × 2 feedback type (success vs. failure). The first variable is between-subject variable, and the last two variables are within-subject variables. Results showed a significant main effect of stimulus type, *F*(1, 50) = 10.52, *p* = 0.002, *η_p_*^2^ = 0.17, BF_Inclusion_ = 5.32, the probe elicited stronger FRN than the irrelevants (5.54 ± 0.56 vs. 4.68 ± 0.45 μV). The interaction between stimulus type and group was significant, *F* (1, 50) = 4.96, *p* = 0.03, *η_p_*^2^ = 0.09, BF_Inclusion_ = 1.60, *Post hoc* tests indicated that the probe elicited a more positive FRN than did the irrelevants in the guilty group (6.63 ± 0.82 vs. 5.17 ± 0.66 μV, *t*(23) = 3.73, *p* = 0.003, Cohen’s *d* = 0.34, BF_10_ = 34.76), while, there was no significant difference between the probe and irrelevants in the innocent group (4.45 ± 0.76 vs. 4.18 ± 0.61 μV, *t*(27) = 0.75, *p* > 0.05, Cohen’s *d* = 0.09, BF_01_ = 3.74). There were no other significant main effects or interaction (*ps* > 0.05). The grand averaged ERPs elicited by feedback stimuli of the group are shown in Figure 3, their PCA-extracted ERP waveforms and scalp distributions are shown in Figures 4A, 5A.

### Feedback-P300 at Pz (390 ms)

Feedback-P300 was analyzed by using the three-way mixed ANOVA of 2 groups (guilty vs. innocent) × 2 stimulus type (probe vs. irrelevant) × 2 feedback type (success vs. failure). The results showed that the main effect of stimulus type was significant, *F* (1, 50) = 14.79, *p* < 0.001, *η_p_*^2^ = 0.23, BF_Inclusion_ = 1.62 × 10^7^, the probe elicited larger feedback-P300 than the irrelevant (3.40 ± 0.76 vs. 1.84 ± 0.69 μV). The main effect of group was also significant, *F* (1, 50) = 7.51, *p* = 0.008, *η_p_*^2^ = 0.13, BF_Inclusion_ = 5.02 × 10^5^, with feedback-P300 in guilty group significantly larger than that in the innocent group (4.53 ± 1.02 vs. 0.71 ± 0.95 μV). The interaction between stimulus type and group was significant, *F* (1, 50) = 21.12, *p* < 0.001, *η_p_*^2^ = 0.30, BF_Inclusion_ = 4.66 × 10^5^. *Post hoc* tests indicated that the probe elicited a more positive feedback-P300 than did the irrelevants in the guilty group [6.25 ± 1.12 vs. 2.82 ± 1.02 μV, *t* (23) = 5.75, *p* < 0.001, Cohen’s *d* = 0.52, BF_10_ = 3.76 × 10^3^]. But among the innocent group, there was no feedback-P300 difference between the probe and irrelevants [0.55 ± 1.03 vs. 0.86 ± 0. 94 μV, *t* (27) = − 0.55, *p* > 0.05, Cohen’s *d* = 0.08, BF_01_ = 4.20]. No other significant main or interaction effects were found (*ps* > 0.05). Brainwave forms of guilty and innocent group in the feedback-P300 are shown in Figure 3, their PCA-extracted ERP waveforms and scalp distributions are shown in Figures 4B, 5B.

### Correlations between self-report and feedback ERPs

To examine whether the detection efficiency of the feedback ERPs (FRN and feedback-P300) was predicted by the extent to which participants believed the feedback, Pearson correlations were conducted. Results showed that there were no significant correlations between the rating scores and feedback-related ERPs in both the guilty group (feedback-P300: *r* = 0.03, *p* > 0.05; FRN: *r* = 0.14, *p* > 0.05), and the innocent group (feedback-P300: *r* = 0.32, *p* > 0.05; FRN: *r* = − 0.03, *p* > 0.05).

### Receiver operating characteristics

**Figure 6:**
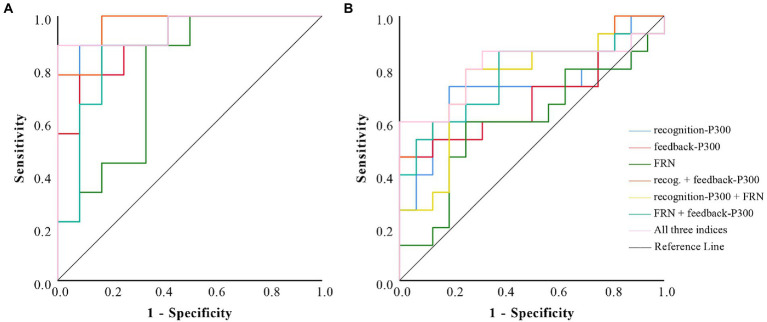
Receiver operating characteristic (ROC) curves of different ERP component indices in the high belief group **(A)** and low belief group **(B)**.

Moreover, to examine the extent to which participants believed feedback influenced the detection efficiency of feedback-related ERPs, we divided participants into two groups based on their rating scores: a high belief group (Mean Scores ± SE = 3.43 ± 0.13, *n* = 21, nine of them are guilty), and a low belief group (Mean Scores ± SE = 1.87 ± 0.06, *n* = 31, 11 of them are guilty). Independent sample *t*-test results showed that there was a significant difference for rating scores between the two groups [*t* (50) = 11.95, *p* < 0.001, 95%CI = [1.30, 1.82], Cohen’s *d* = 2.30]. ROC analyses were then conducted for each group. As predicted, results showed that the feedback-P300 significantly discriminated guilty participants from innocent participants in the high belief groups (AUC = 0.91, 95%CI = [0.78, 1.00], *p* = 0.002), but not in the low belief groups (AUC = 0.68, 95%CI = [0.48, 0.88], *p* = 0.082). Moreover, similar results were found for the FRN, which could significantly discriminate guilty participants from innocent participants in the high belief group (AUC = 0.77, 95%CI = [0.56, 0.97], *p* = 0.039), but not in the low belief group (AUC = 0.60, 95%CI = [0.39, 0.81], *p* = 0.343). Interestingly, this exploratory analysis also showed that the recognition P300 of the high belief group (AUC = 0.94, 95%CI = [0.85, 1.00], *p* = 0.001) was larger than that of the low belief group (AUC = 0.72, 95%CI = [0.52, 0.91], *p* = 0.04). When combining all three indices, the detection efficiency of the high belief group (AUC = 0.95, 95%CI = [0.86, 1.00], *p* < 0.001) was better than that of the low belief group (AUC = 0.81, 95%CI = [0.64, 0.98], *p* = 0.003). For detailed information also see Table 3; Figure 6.

**Table 3:** AUCs and accuracies of various measures of ERP components.

Group	ERP	AUC	95% C.I.
High Belief	Recognition-P300	0.94^**^	0.85–1.00
	feedback-P300	0.91^**^	0.78–1.00
	FRN	0.77^*^	0.56–0.97
	Recog. + feedback-P300	0.96^***^	0.89–1.00
	Recognition-P300 + FRN	0.95^***^	0.86–1.00
	FRN + feedback-P300	0.88^**^	0.73–1.00
	All three indices	0.95^***^	0.86–1.00
Low Belief	Recognition-P300	0.72^*^	0.52–0.91
	feedback-P300	0.68^+^	0.48–0.88
	FRN	0.60	0.39–0.81
	Recog. + feedback-P300	0.80^**^	0.65–0.96
	Recognition-P300 + FRN	0.75^*^	0.56–0.93
	FRN + feedback-P300	0.77^*^	0.59–0.95
	All three indices	0.81^**^	0.64–0.98

+ *p* < 0.10;

* *p* < 0.05;

** *p* < 0.01;

*** *p* < 0.001.

## Discussion

Recently, a novel CIT called the feedback CIT was introduced. This fCIT can use both the recognition P300 and feedback-related ERPs to detect concealed information. Although previous studies showed promising detection efficiency for the fCIT, correctly detecting “guilty” vs. “innocent” participants, the detection efficiency of feedback-related ERPs varies across studies. The present study aimed to replicate previous findings and then examine whether the detection efficiency of feedback-related ERPs was influenced by participants’ belief (or disbelief) in the feedback.

First, we found that probe stimuli elicited a larger FRN and feedback P300 than irrelevants among guilty participants, but this was absent among innocent participants. These results replicated previous findings and suggested that both the FRN and feedback P300 were able to discriminate guilty from innocent at the group level. These results confirmed our hypotheses that guilty participants are motivated to conceal the probe and thus feedback following probe evoked a larger FRN and feedback P300 than feedback following irrelevant (e.g., Sai et al., 2016, 2020; Zheng et al., 2022).

Second, we found that participants’ belief in feedback influenced detection efficiency of feedback-related ERPs, with the detection efficiency of both FRN and the feedback P300 higher in participants in the high belief group compared to the low belief group. Individuals typically use feedback to adjust their subsequent behavior (Hu et al., 2013; Sai et al., 2016). Participants who tended to believe the feedback we provided were more likely to pay more attention to feedback stimuli and then use feedback to adjust their motivation (Luo et al., 2011). For example, when feedback indicated the failure of concealment, guilty participants may have tried harder to conceal the probe in subsequent trials, and thus feedback following probe elicited larger feedback-related ERPs than irrelevants. However, for participants who tended not to believe feedback, they may not have paid much attention to the feedback we provided, and thus no significant difference between probe and irrelevant stimuli was observed for feedback ERPs. Therefore, the discrimination efficiency of the high belief group was better than that of the low belief group. However, future studies should use larger sample sizes to further examine this question.

Additionally, and consistent with many previous findings, we found that the probe stimulus elicited a larger P300 than irrelevants at the group level (for a review, see Rosenfeld et al., 2019). However, it should be noted that we found that the detection efficiency of the recognition P300 was influenced by participants’ belief in feedback, with higher detection efficiency in the high belief group (AUC = 0.94) compared to the low belief group (AUC = 0.72). This is not consistent with our expectation because recognition P300 only reflects the recognition of crime-related information. One possible reason for this result is that participants who tended not believe feedback may have also not paid much attention to the stimuli in general and thus led to a decreased P300 overall, while participants who tended to believe feedback may have invested more attention to the stimuli because they wanted to conceal their knowledge successfully in subsequent trials, and thus leading to higher detection efficiency.

There are several limitations and future directions. First, we found that participants’ belief in feedback influenced detection efficiency of feedback-related ERPs; however, the current sample size is relatively small. Future studies can replicate our results using larger sample sizes to continue to test our conclusions. Second, future research can improve detection efficiency by including methodological manipulations to increase participants’ belief in feedback. For example, the feedback following probe and irrelevant are random (50% accuracy), further studies could make the accuracy of feedback following probe and irrelevant slightly higher than 50%, which may make participants more likely to believe the feedback.

In conclusion, the current study replicated previous findings and showed that feedback related ERPs are able to detect concealed information efficiently. Results also showed that the detection efficiency of feedback related ERP components is affected by the participants’ belief in feedback. The more participants believe in the feedback, the higher the detection efficiency becomes. These findings once again verify the potential of the fCIT to detect concealed information, and provide an improved direction for the CIT.

## Data availability statement

The raw data supporting the conclusions of this article will be made available by the authors, without undue reservation.

## Ethics statement

The studies involving human participants were reviewed and approved by the Ethics Committee of Hangzhou Normal University. The patients/participants provided their written informed consent to participate in this study. Written informed consent was obtained from the individual(s) for the publication of any potentially identifiable images or data included in this article.

## Author contributions

JC, YS, JZ, and LS proposed the study concept and designed the experiments. JC and YS collected and analyzed the data and wrote the draft. JZ participated in data acquisition and analysis. JC, YS, JO, and LS modified the manuscript. LS conceived the idea and wrote the paper. All authors contributed to the article and approved the submitted version.

## Funding

This research was supported by the Natural Science Foundation of China (32071068 to G. Fu, U1736125 to LS) and the Cultivation Project of Provincial Characteristic Key Discipline in the College of Education of Hangzhou Normal University (20JYXK003 to LS).

## Conflict of interest

The authors declare that the research was conducted in the absence of any commercial or financial relationships that could be construed as a potential conflict of interest.

## Publisher’s note

All claims expressed in this article are solely those of the authors and do not necessarily represent those of their affiliated organizations, or those of the publisher, the editors and the reviewers. Any product that may be evaluated in this article, or claim that may be made by its manufacturer, is not guaranteed or endorsed by the publisher.
